# Deficiency of Auxin Efflux Carrier *OsPIN1b* Impairs Chilling and Drought Tolerance in Rice

**DOI:** 10.3390/plants12234058

**Published:** 2023-12-02

**Authors:** Chong Yang, Huihui Wang, Qiqi Ouyang, Guo Chen, Xiaoyu Fu, Dianyun Hou, Huawei Xu

**Affiliations:** College of Agriculture, Henan University of Science and Technology, Luoyang 471000, China; yc051722@163.com (C.Y.); hhwang98@163.com (H.W.); 17538391647@163.com (Q.O.); cguo1010@163.com (G.C.); 13608423875@163.com (X.F.); dianyun518@163.com (D.H.)

**Keywords:** abiotic stress, abscisic acid (ABA), *OsPIN1b*, polar auxin transport, reactive oxygen species (ROS)

## Abstract

Significant progress has been made in the functions of auxin efflux transporter *PIN-FORMED* (*PIN*) genes for the regulation of growth and development in rice. However, knowledge on the roles of *OsPIN* genes in abiotic stresses is limited. We previously reported that the mutation of *OsPIN1b* alters rice architecture and root gravitropism, while the role of *OsPIN1b* in the regulation of rice abiotic stress adaptations is still largely elusive. In the present study, two homozygous *ospin1b* mutants (*C1b-1* and *C1b-2*) were employed to investigate the roles of *OsPIN1b* in regulating abiotic stress adaptations. Low temperature gradually suppressed *OsPIN1b* expression, while osmotic stress treatment firstly induced and then inhibited *OsPIN1b* expression. Most *OsPIN* genes and auxin biosynthesis key genes *OsYUC* were up-regulated in *ospin1b* leaves, implying that auxin homeostasis is probably disturbed in *ospin1b* mutants. The loss of function of *OsPIN1b* significantly decreased rice chilling tolerance, which was evidenced by decreased survival rate, increased death cells and ion leakage under chilling conditions. Compared with the wild-type (WT), *ospin1b* mutants accumulated more hydrogen peroxide (H_2_O_2_) and less superoxide anion radicals $\left(O_{2}^{-}\right)$ after chilling treatment, indicating that reactive oxygen species (ROS) homeostasis is disrupted in *ospin1b* mutants. Consistently, C-repeat binding factor (*CBF*)/dehydration-responsive element binding factor (*DREB*) genes were downregulated in *ospin1b* mutants, implying that *OsDREB* genes are implicated in *OsPIN1b*-mediated chilling impairment. Additionally, the mutation of *OsPIN1b* led to decreased sensitivity to abscisic acid (ABA) treatment in seed germination, impaired drought tolerance in the seedlings and changed expression of ABA-associated genes in rice roots. Taken together, our investigations revealed that *OsPIN1b* is implicated in chilling and drought tolerance in rice and provide new insight for improving abiotic stress tolerance in rice.

## 1. Introduction

In natural habitats, sessile plants are frequently exposed to multiple abiotic stresses, such as cold and drought stresses. These stressors, individually or in combination, potentially attenuate normal biological functions, influence the geographical distribution of plant species and even lead to the death of plants [1,2,3,4,5]. Rice (*Oryza sativa* L.) is one of the most widely cultivated crops in the world and feeds more than half of the world’s population [6,7]. Rice yield stability is tightly associated with environmental conditions, so investigating the molecular mechanism underlying rice responses to environmental constraints is an urgent target through diverse genetic tools.

As one of the most important phytohormones, auxin (indole-3-acetic acid, IAA) plays an essential role in many aspects of plant growth and development [8,9,10,11]. Auxin content and distribution within plant tissues are closely related to polar auxin transport (PAT) [12]. Several auxin influx and efflux carrier protein families are involved in PAT, which, at least, include (AUX1)/LIKE AUX1 (LAX) influx carriers, the PHOSPHOGLYCOPROTEIN (PGP/MDR/ABCB) efflux/influx transporters, the PIN-FORMED (PIN) auxin efflux carriers [12,13] and the structurally similar PIN-LIKES (PILS) [14]. Among these, PIN carriers play a vital role in PAT [15].

AtPIN1 is the first identified auxin efflux carrier in *Arabidopsis* [16] and participates in regulating inflorescence development and root growth by mediating PAT [17]. A total of 12 *OsPIN* genes have been identified in the rice genome and display differential expression profiles in different tissues [18,19,20,21], suggesting their potentially divergent roles in modulating rice growth and development. The *OsPIN1* subfamily contains four *OsPIN1* homologous genes in rice [18,19], and *OsPIN1b* is the homolog of *AtPIN1* and is ubiquitously expressed in rice [20,22]. The primary root length and lateral root number are enhanced by the overexpression of *OsPIN1b* and the down-regulation of *OsPIN1b* reduces adventitious root number and improves tiller numbers [22]. By contrast, the mutation of *OsPIN1b* by CRISPR/Cas9 technology causes no obvious phenotype in rice, only *ospin1a ospin1b* double mutants show pleiotropic phenotypes [23]. Recently, Zhang et al. reported that OsPIN1b participates in regulating leaf inclination in rice [24]. Additionally, nutrient supply influences *OsPIN1b* expression; for example, sufficient nitrate conditions significantly induce *OsPIN1b* expression [25], whereas low-nitrogen and -phosphate conditions significantly down-regulate *OsPIN1b* expression. Further experiments suggested that *OsPIN1b* is involved in the regulation of root architecture under low-nitrogen and -phosphate conditions [26].

Apart from the regulation of growth and development, reports also showed that *OsPIN* genes are differentially expressed under various abiotic stress treatments [20,21], implying that auxin homeostasis mediated by PAT plays a potential role in regulating plant abiotic stress adaptations. Different lines of evidence have suggested that plant chilling adaptation is closely associated with PAT [27,28,29]. An earlier report showed that temperature influences the velocity of exogenous auxin transport in some plant species [30]. Basipetal auxin transport is suppressed by low temperature treatment, while it is restored under room temperature conditions [27]. Auxin content is greatly increased under low temperature conditions [31], and the intracellular trafficking of auxin efflux carriers is inhibited by low temperature treatment [28]. Further research showed that cold tolerance is positively associated with GNOM, a SEC7 containing ARF-GEF, which participates in modulating the endosomal trafficking of auxin efflux carriers [29]. Our previous reports showed that low temperature differentially regulates the expression of *OsPIN* genes in rice roots, among which *OsPIN9* is gradually suppressed under chilling treatment; further experiments indicated that *OsPIN9* negatively regulates rice chilling tolerance [32,33,34], which provides a potential target for breeding cold-resistant crops by CRISPR/Cas9 technology. PAT is also involved in regulating drought tolerance. Drought treatment differentially regulates the transcript abundance of *OsPIN* genes [20,21]. *OsPIN10a* (also designated as *OsPIN3t*) is involved in PAT and the overexpression of *OsPIN10a* increases rice drought tolerance [35], and the loss of function of *OsPIN2* leads to impaired drought tolerance in rice [36]. Additionally, *OsPIN* genes are implicated in heavy metal stress. The overexpression of *OsPIN2* alleviates aluminum-induced cell damage in rice root tips [37], which is realized by elevating endocytic vesicular trafficking and aluminum internalization [38]. Other reports also showed that the expression of *OsPIN* genes is differentially regulated by cadmium and arsenic treatments [39,40], and the mutation of auxin influx carrier *OsAUX1*, which is involved in the regulation of PAT, increases the sensitivity to cadmium treatment in rice [41]. Taken together, auxin homeostasis plays a potential function in regulating abiotic stress adaptations, which is probably mediated by the regulation of PAT.

Abiotic factors, including cold and drought stresses, are crucial determinants for the growth and development of many crop species and negatively influence crop productivity [3,42]. Therefore, investigating the molecular mechanism underlying plant response to abiotic stresses is crucial for food security worldwide. Plant hormones, such as abscisic acid (ABA), auxin, ethylene (ET), gibberellic acid (GA), brassinosteroid (BR), jasmonic acid (JA), salicylic acid (SA) and strigolactone (SL), are key plant growth regulators and play pivotal roles in response to various environmental stresses [43]. Typically, ABA is considered one of the main phytohormones involved in water deficiency for the induction of stomatal closure during water stress [44]. Conversely, SA, JA and ET levels increase upon pathogen infection and are regarded as the major phytohomones in biotic stress adaptations [45]. As the first discovered plant hormone, auxin plays a key role in regulating virtually all aspects of plant growth and development, as well as adapting to various abiotic stresses [46]. To date, auxin has been proven to be implicated in adapting to high temperature [47,48,49], salinity [50,51], drought [51,52], heavy metals [53] and low temperature [54]. Besides phytohormones, more and more genes were reported to participate in regulating abiotic stress adaptations, such as wheat *GLUTATHIONE PEROXIDASE* (*TaGPX1-D*) and *SODIUM*/*CALCIUM EXCHANGER-LIKE* (*TaNCL2-A*) genes, which were reported to be implicated in salinity and osmotic stress tolerance [55,56]. Although significant progress has been made in understanding the role of auxin in regulating plant adaptation to adverse environmental factors [57], the fundamental molecular mechanism underlying auxin regulating chilling response is still elusive. In a previous study, we reported that the mutation of *OsPIN1b* alters plant architecture and root gravitropism [58]. However, little is known about the role of *OsPIN1b* in abiotic stress adaptations. Here, we observed that *OsPIN1b* is differentially regulated by chilling and osmotic stress treatments, and further investigation showed that the mutation of *OsPIN1b* impairs cold and drought tolerance, demonstrating that PAT mediated by OsPIN1b plays a crucial role in abiotic stress adaptations.

## 2. Results

### 2.1. Expression Profiles of OsPIN1b under Chilling and Osmotic Stress Conditions

**Figure 1:**
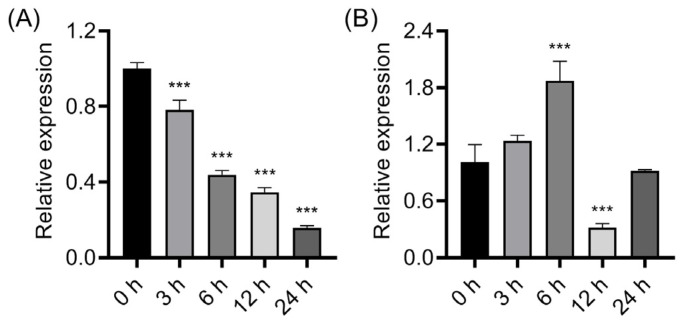
Expression profile analysis of *OsPIN1b* under chilling (**A**) or 20% PEG6000 (**B**) conditions. Values are means ± standard deviation (SD) (n = 3). Data were analyzed via ANOVA and Tukey’s tests at *p* < 0.05 significance level. ***: *p* < 0.001.

To investigate the role of *OsPIN1b* in abiotic stresses, quantitative real-time PCR (qRT-PCR) was performed to assess the expression of *OsPIN1b* under chilling and osmotic stress conditions. The 14-day-old seedlings were treated with low temperature (4 °C) or 20% PEG6000, and leaves were sampled at indicated time points for *OsPIN1b* expression analysis using qRT-PCR. The results revealed that low temperature gradually suppressed the expression of *OsPIN1b* and that *OsPIN1b* was decreased by 22% after chilling for 3 h and progressively decreased by 84% after chilling for 24 h (Figure 1A). By contrast, *OsPIN1b* transcript abundance was firstly induced and then suppressed by PEG6000 treatment (Figure 1B). These results suggested that *OsPIN1b* has a potential role in regulating chilling and osmotic stress adaptations.

### 2.2. Expression Analysis of OsPIN and OsYUC Genes in ospin1b Mutants

**Figure 2:**
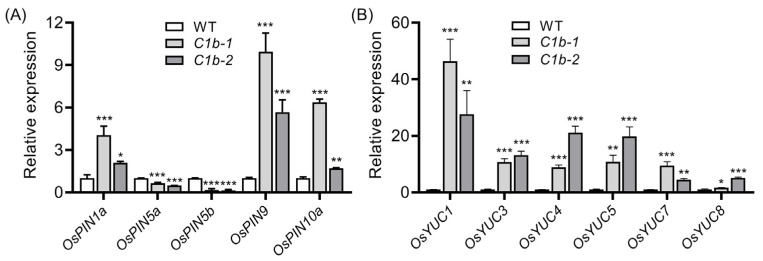
Expression analysis of auxin efflux genes *OsPIN* (**A**) and auxin biosynthesis genes *OsYUC* (**B**) in wild-type (WT) and *ospin1b* leaves. Values are means ± standard deviation (SD) (n = 3). Data were analyzed via ANOVA and Tukey’s tests at *p* < 0.05 significance level. *: *p* < 0.05; **: *p* < 0.01; ***: *p* < 0.001.

Previously, we have reported that the mutation of *OsPIN1b* disturbs auxin homeostasis, alters rice architecture and root gravitropism in rice [58]. PIN family proteins play a vital role in PAT [15], and the *YUCCA* (*YUC*) gene family, which encodes flavin monooxygenase-like enzymes, is widely accepted as the key auxin biosynthesis gene family in plants [59,60,61]. We wondered whether the mutation of *OsPIN1b* influences other *OsPIN* genes, as well as *OsYUC* genes expression. To this end, two homozygous *ospin1b* mutants *ospin1b-1* (*C1b-1*) and *ospin1b-2* (*C1b-2*), which were previously generated using CRISPR/Cas9 technology [58], were employed for further investigation. qRT-PCR analysis showed that several *OsPIN* genes, including *OsPIN1a*, *OsPIN9* and *OsPIN10a*, were significantly induced, while *OsPIN5a* and *OsPIN5b* were suppressed in *ospin1b* leaves when compared with wild-type (WT) plants (Figure 2A). These results indicated that the mutation of *OsPIN1b* probably disrupts PAT and consequently perturbs auxin homeostasis. By contrast, the mutation of *OsPIN1b* greatly enhanced the expression of *OsYUC* genes. In detail, *OsYUC1* increased 27–45 fold, followed by *OsYUC3*, *OsYUC4* and *OsYUC5*, which displayed a great enhancement of about 9–20 fold (Figure 2B). 

### 2.3. Mutation of OsPIN1b Substantially Impairs Rice Chilling Tolerance

**Figure 3:**
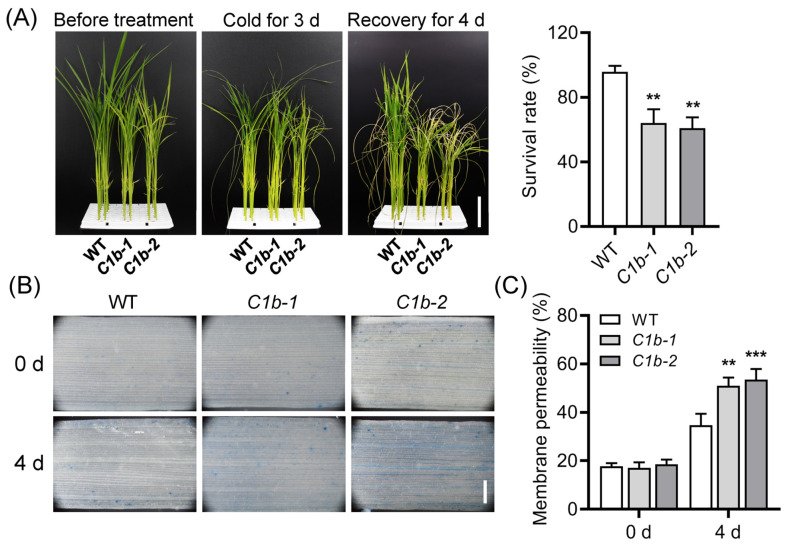
Mutation of *OsPIN1b* impairs rice chilling tolerance. (**A**) The 14-day-old seedlings were treated in 4 °C for 3 days followed by 4-day recovery. Bar = 4 cm. (**B**) Trypan blue staining. Bar = 1 mm. (**C**) Electrolyte leakage. Values are means ± standard deviation (SD) (n = 6). Data were analyzed via ANOVA and Tukey’s tests at *p* < 0.05 significance level. **: *p* < 0.01; ***: *p* < 0.001.

PAT is probably implicated in plant cold adaptation [28]. Recently, we reported that OsPIN9, another auxin efflux carrier, is involved in regulating rice chilling tolerance by modulating ROS homeostasis [33,34]. However, whether *OsPIN1b* is involved in cold adaptation is still elusive. To this end, 14-day-old seedlings were transferred from 28 °C to 4 °C for 3 days and followed by another 4-day recovery under normal conditions. After chilling for 3 days, almost all *ospin1b* leaves displayed a clearly wilted phenotype, whereas the WT leaves showed a relatively normal phenotype. After recovery, the survival rate of *ospin1b* was significantly decreased by 33–36% when compared with that of WT plants (Figure 3A). 

Low temperature damages plant cells and even causes plant death [5]. Trypan blue staining was performed to detect cell death. The staining was slightly more intensive in *ospin1b* mutants than in WT plants under normal conditions, while it was obviously stronger in *ospin1b* leaves than in WT plants under chilling stress conditions (Figure 3B). These results suggested that more cell death occurred in *ospin1b* mutants than in WT plants after chilling treatment. Membrane permeability, which is evaluated through the measurement of electrolyte leakage in rice leaves, was comparable with the WT plants in *ospin1b* mutants under normal conditions, while, after chilling treatment, it was significantly increased in *ospin1b* leaves compared with WT plants (Figure 3C). Collectively, these results strongly suggest that the mutation of *OsPIN1b* indeed impairs rice chilling tolerance.

### 2.4. Mutation of OsPIN1b Disrupts ROS Homeostasis under Chilling Stress Conditions

**Figure 4:**
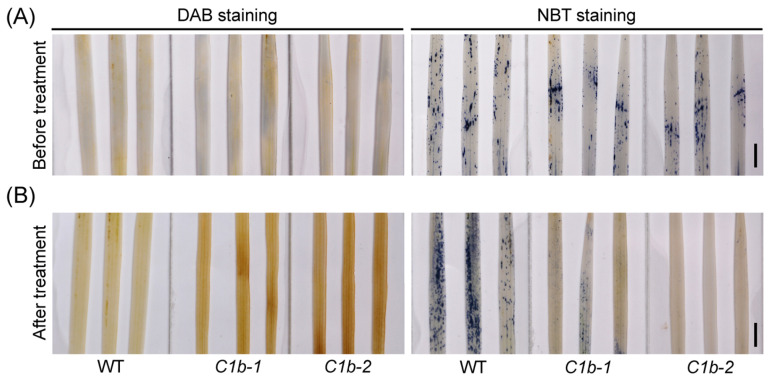
DAB and NBT staining in wild-type (WT) and *ospin1b* mutants before (**A**) and after (**B**) cold stress. Bar = 0.5 cm. At least three independent experiments with 6 seedling leaves per experiment were performed.

Plant chilling tolerance is tightly associated with ROS homeostasis in plant cells [33,62]. *ospin1b* mutants are more sensitive to chilling stress; we wondered whether the chilling treatment influences ROS homeostasis in *ospin1b* mutants. 3,3′-diaminobenzidine (DAB) and nitro blue tetrazolium (NBT) staining were performed to evaluate hydrogen peroxide (H_2_O_2_) and superoxide anion radicals $\left(O_{2}^{-}\right)$ content in rice leaves, respectively. Before chilling treatment, the DAB staining of *ospin1b* leaves was comparable with that in WT plants, and the NBT staining of *ospin1b* leaves was slightly lighter than in WT plants (Figure 4A). Conversely, cold treatment obviously induced the accumulation of H_2_O_2_ and $O_{2}^{-}$ in WT leaves. Compared with the WT, *ospin1b* leaves accumulated more H_2_O_2_ under chilling conditions. To our surprise, NBT staining still displayed an obvious lighter intensity than in WT plants even after chilling treatment (Figure 4B). Together, the ROS homeostasis is perturbed in *ospin1b* mutants after cold treatment, which probably contributes to the impaired chilling tolerance in *ospin1b* mutants.

### 2.5. CBF/DREB Regulon Is Implicated in Chilling Tolerance in ospin1b Mutants

**Figure 5:**
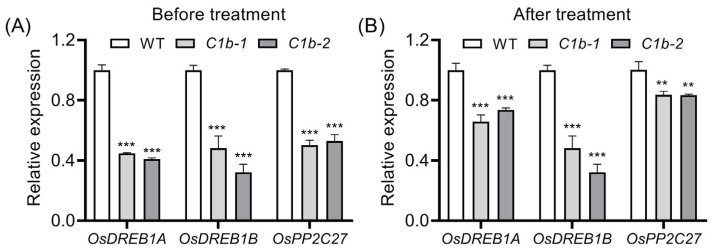
Expression analysis of *OsDREB1A*, *OsDREB1BI* and *OsPP2C27* before (**A**) and after (**B**) chilling treatments. Values are means ± standard deviation (SD) (n = 3). Data were analyzed via ANOVA and Tukey’s tests at *p* < 0.05 significance level. **: *p* < 0.01; ***: *p* < 0.001.

The well-known C-repeat binding factor (CBF)/dehydration-responsive element binding factor (DREB)/cold-regulated (COR) regulon plays a crucial role in plant low-temperature adaptation [63,64,65]. The mutation of *OsPIN1b* led to decreased chilling tolerance in rice (Figure 3). To understand the molecular basis of this regulatory mechanism upon chilling stress, we detected the expression of *OsDREB1A* [66,67], *OsDREB1B* [67,68] and *PROTEIN PHOSPHATASE 2C* (*OsPP2C27*) [69], which has been proved to be involved in rice chilling tolerance, in 14-day-old seedling leaves before and after chilling for 8 h. The results showed that the mutation of *OsPIN1b* clearly decreased the expression of these genes before chilling treatment (Figure 5A). After chilling treatment, the expression of *OsDREB1A*, *OsDREB1B* and *OsPP2C27* in *ospin1b* mutants was still significantly lower than in WT plants (Figure 5B); these results indicated that these *DREB*/*COR* genes are implicated in chilling tolerance in *ospin1b* mutants.

### 2.6. Loss of OsPIN1b Function Leads to Enhanced Resistance to ABA Treatment

**Figure 6:**
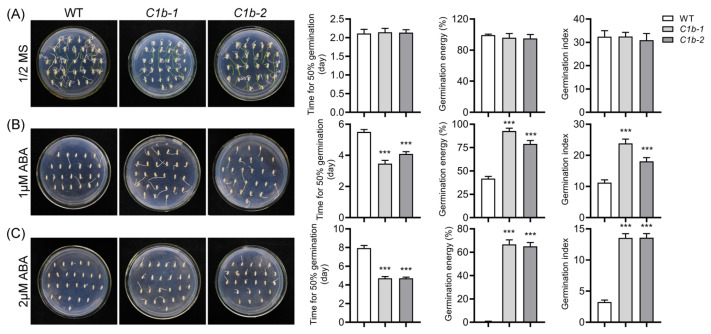
Mutation of *OsPIN1b* influences ABA response. The wild-type (WT) and *ospin1b* seeds were cultured in 1/2 MS medium (**A**), 1/2 MS medium added 1 μM ABA (**B**) and 1/2 MS medium added 2 μM ABA (**C**). The photos were shot after germination for 5 d. Values are means ± standard deviation (SD) (n = 3). Data were analyzed via ANOVA and Tukey’s tests at *p* < 0.05 significance level. ***: *p* < 0.001.

More and more evidence shows that auxin homeostasis is tightly associated with ABA metabolism [36,70]. Since the loss of function of *OsPIN1b* disturbed auxin homeostasis in rice (Figure 2) [58], we asked if the disturbing of auxin homeostasis in *ospin1b* mutants influences sensitivity to ABA treatment. To this end, we evaluated the sensitivity of WT and *ospin1b* seeds to ABA treatment. Three different indicators, time to 50% germination, germination energy and germination index, were employed to monitor rice seed germination ability. There was no significant difference in germination ability between the WT and *ospin1b* mutants when cultured in half-strength Murashige and Skoog (MS) medium (Figure 6A). As expected, 1 μM ABA treatment obviously suppressed WT seed germination, and 2 μM ABA treatment almost completely inhibited the germination of the WT seeds. In contrast, *ospin1b* mutants showed more tolerance to ABA treatment than WT. The time to 50% germination of *ospin1b* mutants was significantly lower than that of WT plants, and the germination energy and germination index of *ospin1b* mutants were dramatically higher than those of WT plants, especially under 2 μM ABA treatment conditions (Figure 6B,C). These data strongly suggested that the impairment of *OsPIN1b* enhances the resistance to ABA treatment, implying that the ABA pathway is probably perturbed in *ospin1b* mutants.

### 2.7. Mutation of OsPIN1b Decreases Drought Tolerance

**Figure 7:**
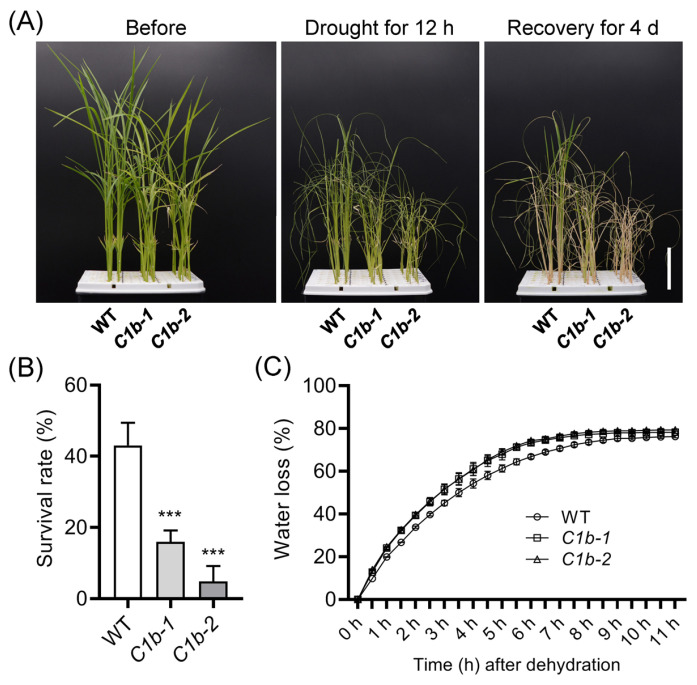
Mutation of *OsPIN1b* impairs rice drought tolerance. The 14-day-old seedlings were exposed to air for 12 h followed by another 4-day recovery. Bar = 4 cm (**A**), and then the survival rate was assessed statistically (n = 24). Data were analyzed via ANOVA and Tukey’s tests at *p* < 0.05 significance level. ***, *p* < 0.001 (**B**). Water loss of detached leaves was analyzed every 30 min (**C**). Values are means ± standard deviation (SD) (n = 3).

As we know, ABA plays a vital role in abiotic stress responses, especially in drought stress response [71]. Due to the decreased sensitivity of *ospin1b* mutants to ABA treatment in seed germination, we further assayed the drought tolerance of *ospin1b* mutants. A withholding water experiment was performed to assess the drought tolerance of *ospin1b* seedlings [36]. The 14-day-old seedlings were exposed to air for 12 h, and then moved back to hydroponic solution for 4-day recovery. The 12-h treatment seriously influenced the growth of rice seedlings; almost all plant leaves were wilted (Figure 7A). After 4-day recovery, nearly half of WT plants survived, whereas almost all *ospin1b* mutants were dead. The survival rate of *ospin1b* mutants was significantly reduced by 27–38% compared with that of WT plants (Figure 7B). 

The water loss of detached leaves is a key indicator to assess plant drought tolerance [72,73]. We then monitored the rate of water loss in leaves detached from 14-day-old plants every 30 min. The fresh weight of the detached leaves was measured over 11 h. *ospin1b* leaves showed a faster water loss than WT leaves after dehydration for 30 min, and kept a higher water loss rate than WT plants during the whole measuring time (Figure 7C). These results indicated that the impaired drought tolerance is, at least in part, caused by the higher water loss rate in *ospin1b* mutants.

For further confirming the drought sensitivity of *ospin1b* mutants, PEG6000 treatment, which causes osmotic stress, was employed to mimic drought treatment and analyze the osmotic stress tolerance of WT and *ospin1b* mutants. The 4-day-old seedlings were subjected to 0% or 20% PEG6000 treatment for 12 days [74]. The PEG6000 treatment obviously retarded rice growth, especially inhibiting shoot growth. In comparison with the WT, the shoot height of *ospin1b* mutants decreased by 14–18% under normal conditions (Figure S1A), whereas it dramatically decreased by 39–41% after the 12-day osmotic stress treatment (Figure S1B). Conversely, the root length of *ospin1b* mutants showed a similar decrease compared with WT plants both before and after osmotic stress treatments (Figure S1). Consistently, the relative shoot height of *ospin1b* mutants was more greatly declined than that of WT after the 12-day PEG treatment, while the relative root length was comparable with that of WT (Figure S2). Collectively, these results strongly indicated that the loss of function of *OsPIN1b* substantially impairs rice drought tolerance, which was mimicked by the PEG6000 treatment. 

### 2.8. Mutation of OsPIN1b Affects the Expression of ABA-Associated Genes

**Figure 8:**
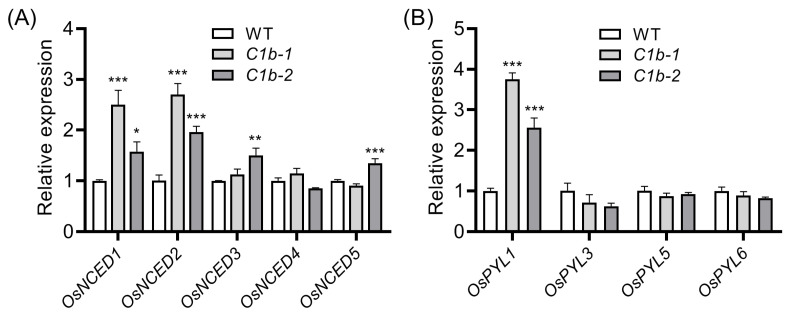
Relative expression of ABA biosynthesis genes (**A**) and ABA acceptor genes (**B**) in wild-type (WT) and *ospin1b* roots. Data are means ± SD and analyzed via ANOVA and Tukey’s tests at *p* < 0.05 significance level. *: *p* < 0.05; **, *p* < 0.01, ***, *p* < 0.001.

Since the mutation of *OsPIN1b* resulted in decreased ABA sensitivity and impaired drought tolerance (Figure 6 and Figure 7), we therefore asked whether the expression of ABA-associated genes is influenced in *ospin1b* mutants. The 9-cis-epoxycarotenoid dioxygenase (NCED) family is reported to be the rate-limiting enzymes for ABA biosynthesis [75], and the Pyrobactin Resistance/Pyrobactin 1-Like/Regulatory Components of the ABA Receptor (PYR1/PYLs/RCARs) protein family are widely regarded as ABA acceptors and play key roles in ABA signaling [76]. The expression of *OsNCED* and *OsPYL* genes was analyzed in 7-day seedling roots by qRT-PCR. The results showed that *OsNCED1* and *OsNCED2* were significantly induced in *ospin1b* mutants (Figure 8A), whereas only *OsPYL1* displayed a significant increase in *ospin1b* mutants (Figure 8B). 

## 3. Discussion

A total of 12 *OsPIN* genes have been identified in the rice genome [18,19]; previous reports have investigated the expression profiles of *OsPIN* genes under various phytohormones and abiotic stress treatments [18,20,21]. As auxin efflux carriers, IAA treatment could induce almost all *OsPIN* genes’ expression [18,21]. The mutation of *OsPIN1b* influences other *OsPIN* genes’ expression (Figure 2A), and *OsPIN2*, *OsPIN5b* and *OsPIN10a* were reported to be involved in the regulation of auxin transport [35,36,77]. These results suggested that IAA and *OsPIN* genes could affect each other to regulate plant growth and development. PIN is associated with abiotic stress adaptation [33,34,35,36], and abiotic stress treatments influence *OsPIN* genes’ expression. Cold stress inhibited the expression of *OsPIN1c*, *OsPIN1d*, *OsPIN9*, *OsPIN10* and *OsPIN10b* in rice roots, and paralogous *OsPIN* genes displayed a similar expression profile under cold stress [34], implying that they might act synergistically to modulate rice cold tolerance. Heat stress mainly suppresses *OsPIN* genes’ expression, while salt and drought treatments mainly induce the expression of *OsPIN* genes [21], indicating that PAT mediated by OsPIN carriers has a potential role in regulating abiotic stress tolerance. Previously, we have indicated that *OsPIN1b* is involved in rice architecture alteration and root gravitropism [58], while the role of *OsPIN1b* in regulating abiotic stress response is largely unknown. In this study, we observed that *OsPIN1b* expression was progressively inhibited by low temperature in rice leaves (Figure 1A). Further investigation demonstrated that the loss of function of *OsPIN1b* impairs rice chilling tolerance (Figure 3). In contrast, *OsPIN9* is also suppressed by low temperature, while *ospin9* mutants own a chilling-resistant phenotype [33]. Expression analysis showed that the mutation of *OsPIN1b* greatly induced the expression of *OsPIN9* (Figure 2A), and the overexpression of *OsPIN9* decreases rice chilling tolerance [34]; it is reasonable to consider that the impaired chilling tolerance of *ospin1b* mutants might partly be caused by the improved expression of *OsPIN9* in *ospin1b* mutants. Besides *OsPIN9*, we also noticed that several *OsPIN* genes were also differentially expressed in *ospin1b* mutants (Figure 2A), indicating that OsPIN carriers act cooperatively in regulating PAT. 

Intriguingly, *ospin1b* mutants derived from different studies display various phenotypes. We observed that the mutation of *OsPIN1b* causes pleiotropic phenotypes in rice [58]. Consistently, the down-regulation of *OsPIN1b* by RNAi or T-DNA insertion also results in growth retardation in rice [22,26]. However, reports also showed that *ospin1* single mutants created by CRISPR/Cas9 have no dramatic phenotypes and only *pin1a pin1b* double mutants display obvious phenotype alteration [23]. An explanation of this difference might be that the newly formed OsPIN1b protein created by CRISPR/Cas9 is still partially functional. CRISPR/Ca9 technology usually causes small insertions or deletions at specific points in target genes [78], which results in various mutation types and possible phenotype variations. For example, CRISPR/Cas9 constructs targeting various exons of the *Waxy* gene, which encodes a granule-bound starch synthase (GBSS) and plays a crucial role in regulating amylose synthesis in the endosperm, were transformed into rice and generated edited lines with different amylose content [79,80], further analysis showed that the mutation of the *Waxy* promoter by CRISPR/cas9 could also produce mutant lines exhibiting different amylose contents [81]. Additionally, the paralogous gene *OsPIN1a* can partly complement the loss of function of *OsPIN1b* [23], which could partially rescue the retarded growth caused by the mutation of *OsPIN1b*. Finally, an off-target event cannot be completely ruled out in *ospin* mutants created by CRISPR/Cas9 technology in our study, despite the fact that the potential off-target sites were analyzed by Sanger sequencing and no mutations are found in *ospin1b* mutants [58]. 

Over the past few years, the mechanism underlying plant responses to chilling stress has been extensively investigated [82,83,84]. For example, the CHILLING TOLERANCE DIVERGENCE 1 (COLD1)/G-protein α subunit 1 (RGA1) complex, which functions as a plant cold sensor, was demonstrated to play a key role in rice cold tolerance [63,85]. Ca^2+^ channels play a substantial role in cold response, the *CYCLIC NUCLEOTIDE-GATED CHANNEL* (*CNGC*) genes respond differentially to low temperature [86], and the overexpression of *OsCNGC9* was proven to confer enhanced chilling tolerance in rice [66]. Additionally, ROS [87,88] and phytohormones [89,90] were also reported to be implicated in chilling tolerance. In particular, the well-known *CBF*/*DREB*-dependent transcriptional regulatory genes play a central role in chilling tolerance [5,89,91]. The expression of *OsDREB* genes is dramatically induced by low temperatures [67,68], and the overexpression of *OsDREB1A* or *OsDREB1B* enhances chilling tolerance in rice [65,67]. The mutation of *OsPIN1b* decreased the expression of *OsDREB1A* and *OsDREB1B* before and after chilling treatments (Figure 5), indicating that the impaired chilling tolerance of *ospin1b* mutants is, at least in part, attributed to the decreased expression of *OsDREB1A* and *OsDREB1B*. It was reported that cold-induced *OsPP2C27* directly dephosphorylates phospho-OsMAPK3 and phospho-OsbHLH002 and decreases rice chilling tolerance by suppressing the expression of *TREHALOSE-6-PHOSPHATE PHOSPHATASE1* (*OsTPP1*) and *OsDREBs*, indicating that OsPP2C27 negatively regulates rice chilling tolerance [69]. In agreement with this, we observed that *OsPP2C27* expression increased to a higher extent in *ospin1b* mutants than in WT after chilling treatment (Figure 5), which might also contribute to the impaired chilling tolerance of *ospin1b* mutants.

ROS serve as key signaling molecules in regulating numerous biological processes and respond to fluctuating environmental cues, while ROS levels must be finely controlled in cells by many ROS scavenging and detoxification systems [92,93]. Abiotic stress factors can induce the production of ROS. At the early stress stage, low level ROS are regarded as signals to trigger various stress-responsive activities, while ROS accumulation, usually occurring at the later stress stage, damages plant cells and even causes cell death [82]. It was reported that chilling-tolerant *japonica* varieties accumulate ROS earlier than chilling-sensitive *indica* varieties [88]. Accumulating evidence suggests that ROS homeostasis plays an important role in chilling adaptation [4,82,91]. Previously, we reported that the loss of the function of *OsPIN9* enhances rice chilling tolerance, and *ospin9* mutants accumulate more and less ROS than WT at the early and later chilling stages, respectively [33]; suggesting that ROS homeostasis plays a crucial role in modulating chilling tolerance. Most recently, we further demonstrated that the over-exprssion of *OsPIN9* impairs rice chilling tolerance, and the accumulation of H_2_O_2_ instead of $O_{2}^{-}$ was observed in *OsPIN9*-overexpressing rice plants after chilling treatment [34], suggesting that it is H_2_O_2_ rather than $O_{2}^{-}$ which probably damages plant cells. In line with this, the mutation of *OsPIN1b* also confers chilling sensitivity in rice (Figure 3) and only H_2_O_2_ accumulation was detected in *ospin1b* mutants after chilling treatment (Figure 4). By contrast, many studies showed that low temperature usually triggers the accumulation of both H_2_O_2_ and $O_{2}^{-}$, which leads to the damage of cells. For example, ethylene-responsive factors *PtrERF9* and *ERF108* from trifoliate orange (*Poncirus trifoliata* (L.) Raf.) are involved in regulating freezing tolerance, and the knockdown of these two genes results in the simultaneous accumulation of H_2_O_2_ and $O_{2}^{-}$ after chilling treatment [94,95]. These results implied that the mechanism underlying ROS homeostasis mediated by *OsPIN* genes during chilling stress is unique and needs further investigation.

It was reported that the exogenous application of auxin can induce the production of H_2_O_2_ in rice leaves [33,70], indicating that auxin level affects H_2_O_2_ content. Further investigation demonstrated that auxin can regulate ROS production by transcriptionally regulating transcription factor *ROOT HAIR DEFECTIVE SIXLIKE4* (*RSL4*) [96]. *OsPIN* and *OsYUCCA* genes function in auxin transport and biosynthesis, respectively, and the expression of *OsPIN* and *OsYUC* genes was differentially influenced in *ospin1b* mutants (Figure 2), implying that auxin homeostasis is probably disrupted in *ospin1b* mutants, as reported previously [58]. It was reported that the expression of *OsYUC* genes is negatively regulated by auxin [59,97], so the improved expression of *OsYUC* genes in *ospin1b* mutants (Figure 2B) implied that the auxin level in *ospin1b* leaves is probably decreased compared with that in WT plants. Consistently, the overexpression of *OsPIN9* results in the decrease in auxin in rice leaves [98], and the mutation of *OsPIN1b* notably induced the expression of *OsPIN9* (Figure 2A). It is reasonable to propose that the auxin level in *ospin1b* might be decreased, while it still needs further experimental confirmation.

Traditionally, auxin and ABA are considered to play crucial roles in regulating growth and stress adaptations, respectively [99]. However, accumulating evidence suggested that auxin and ABA can interact with each other and influence many aspects of plant growth and development [100]. For example, the mutation of *YUC1* and *YUC2* leads to the deficiency of the IPyA pathway and to resistance upon ABA treatment during seed germination, while auxin accumulation causes hypersensitivity to ABA in seed germination analysis [101]. PAT is also closely associated with ABA homeostasis [36]. The mutation of *AUX1* or *PIN2* in *Arabidospsis* displays an ABA-resistant phenotype in seed germination assays [102]. However, *ospin2* mutants are more sensitive to ABA treatment in seed germination [36], implying that the underlying mechanism in auxin–ABA interactions in monocot and dicot plants is probably different. In the present study, *ospin1b* mutants are more resistant to ABA treatment than WT plants (Figure 6), and the transcript of ABA-related genes was differentially expressed in *ospin1b* mutants (Figure 8); these results indicated that the mutation of *OsPIN1b* probably perturbs ABA homeostasis. 

ABA plays a prominent role in controlling stomatal aperture [103] and ABA level is tightly associated with drought stress tolerance [104,105]. Apart from ABA, H_2_O_2_ and Ca^2+^ also function as key signaling molecules involved in controlling stomatal closure [103]. Although ABA content is absent in this study, several lines of indirect evidence are presented to prove the possible decreased ABA level in *ospin1b* mutants. First, *ospin1b* mutants are more resistant to ABA treatment than WT plants (Figure 6). Second, the water loss rate of *ospin1b* was higher than that of WT plants (Figure 7C). Third, *OsPYL1* expression is dramatically suppressed by ABA treatment [106], indicating that *OsPYL1* expression is closely associated with ABA level. In our study, *OsPYL1* was strikingly induced in *ospin1b* mutants (Figure 8B), implying decreased ABA content in *ospin1b* mutants. In line with this, the withholding water assay demonstrated that the survival rate of *ospin1b* was significantly decreased compared with that of WT plants (Figure 7B), and the osmotic stress experiment showed that seedling shoots of *ospin1b* were more sensitive to PEG6000 treatment compared to those of WT plants (Figure S1). These results strongly suggested that the mutation of *OsPIN1b* confers drought sensitivity in rice. 

It is well known that drought stress induces the synthesis of ABA and triggers stomatal closure, indicating that stomatal closure is tightly associated with ABA level and drought stress [107]. Correspondingly, the water loss rate is usually regarded as an important physiological indicator to assess plant drought tolerance. For example, a previous report showed that the overexpression of *OsGH3-2*, encoding an IAA-amino-acid-conjugating enzyme, results in a reduced ABA level, faster water loss and more opened stomata [70]. However, the water loss under drought stress is not only related to stomatal closure. Other reports showed that the overexpression of *ABSCISIC ACID-INSENSITIVE LIKE-2* (*OsABIL2*) leads to ABA insensitivity, increased water loss and increased stomatal density in rice [108]. These findings suggested that water loss is not only associated with stomatal closure but also stomatal density, both of which are regulated by ABA level. We observed that the water loss rate is increased in *ospin1b* mutants (Figure 7C), while the underlying mechanism is not clear. Intriguingly, *ospin2* [36] and *ospin1b* mutants are sensitive and resistant to ABA treatment, respectively, whereas both of the two mutants showed a drought-sensitive phenotype (Figure 7) [36], suggesting that the mechanism underlying *OsPIN* genes’ participation in modulating crosstalk between auxin and ABA, as well as drought adaptation, needs further investigation. Collectively, the loss of *OsPIN1b* function resulted in the disruption of ABA homeostasis, and possibly caused a decrease in ABA content in rice, which accounts for the increased water loss under drought stress conditions and, finally, the impaired drought tolerance. However, the underlying molecular mechanism of *OsPIN1b* in regulating ABA homeostasis is still largely unknown.

## 4. Materials and Methods

### 4.1. Plant Materials and Abiotic Stress Treatments

Rice *japonica* variety Nipponbare and *ospin1b* mutants [58] were employed for the experiments. Rice plants were cultured using hydroponic systems according to our previous report [24]. In brief, after sterilization, rice seeds were incubated in Petri dishes with wetted filter papers at 28–30 °C for several days in the dark. The germinated seeds were moved to Kimura B complete nutrient solution in plant growth chambers with a cycle of 12 h light/12 h dark (30 °C/25 °C) and 60–80% relative humidity.

To analyze the transcriptional profile of *OsPIN1b* to low-temperature and drought treatment, the 14-day-old seedlings were treated with low temperature (4 °C) or osmotic stress (20% PEG6000), and leaves were sampled at the indicated time point for gene expression analysis. The experiments were repeated three times independently and each expression profile was verified in triplicate.

For chilling treatment, the 14-day-old seedlings cultured in the Kimura B solution were transferred to 4 °C conditions for 3 days followed by 4-day recovery, and then the survival rate and related physiological indicators were analyzed. For drought tolerance evaluation, withholding water analysis was performed according to previous report [36]. Briefly, the *ospin1b* mutants and WT controls were exposed to air for 12 h, and then transferred to a hydroponic solution for recovery. The survival rate analysis was performed according to a previous report [109]. Briefly, plants displaying relatively normal growth with at least one green leaf were regarded as living, whereas those with withered leaves, especially those without green young leaves, were classified as dead. Additionally, 20% PEG6000 treatment was employed to mimic drought stress as in previous report [74]. Each treatment was performed independently at least three times.

### 4.2. RNA Extraction and Quantitative Real-Time PCR Analysis

Total RNA was extracted using RNAiso Plus (TAKARA Bio Inc., Dalian, China), and reverse transcription was conducted using RT ProMix for qPCR (+gDNA clearer) (Guangzhou CISTRO Biotech Company, Ltd., Guangzhou, China). qPCR was performed using 2× Ultra SYBR Green qPCR Mix (Guangzhou CISTRO Biotech Company, Ltd., Guangzhou, China) and Lightcycler^®^ 96 system. For detecting the expression of target genes in WT and *ospin1b* mutants, the relative expression level of target genes in WT was defined as 1. For analyzing *OsPIN1b* response to abiotic stress treatments, the relative expression level of *OsPIN1b* before treatment was set as 1. The primers for qRT-PCR are listed in Table S1, and the gene names and ID numbers used for qRT-PCR in this study are presented in Table S2.

### 4.3. Determination of Physiological Indicators

Trypan blue, DAB and NBT staining were performed according to our previous report with minor modifications [33]. Briefly, for the trypan blue staining, leaves were sampled and incubated with a lactophenol-trypan blue solution (10 mL lactic acid, 10 mL glycerol, 10 g phenol and 10 mg trypan blue, mixed in 10 mL distilled water), and then boiled in a water bath for 10 min followed by 1 h at room temperature. Chloral hydrate solution (25 g chloral hydrate dissolved in 10 mL distilled water) was used for decolorization. For DAB and NBT staining, leaves were incubated in 1 mg/mL DAB and 1 mg/mL NBT solution, respectively, at −0.1 Mpa for 30 min, and then the leaves were decolorized with 95% alcohol. 

Membrane permeability was evaluated by relative electrolyte conductivity (R1/R2). The top fully expanded leaves were collected and washed with ddH_2_O several times, and then cut into sections (about 1 cm in length) and infiltrated with ddH_2_O at −0.1 Mpa for 30 min. The conductance of the samples in 20 mL ddH_2_O for 3 h (R1), and after boiling for 30 min (R2), was measured.

Water loss rates were measured using 14-day-old seedling leaves. In brief, rice leaves were sampled and immediately placed in a dry plastic bag in an icebox. The leaves were weighted at the time point indicated in Figure 7C.

### 4.4. ABA Treatment Assays 

ABA sensitivity during seed germination was performed following previous report [36]. At least 24 de-husked seeds per replicate of *ospin1b* mutants and WT Nipponbare were firstly sterilized and then placed on 1/2 MS medium plates supplemented with 0, 1 or 2 μM ABA for germination analysis. Germination ability was assayed daily. If the radicle protruded about 2 mm through the seed coat, the seed was considered to have germinated. Three traits of seed vigor, including time for 50% germination, germination energy and germination index, were tested according to Fu et al. [110] and He et al. [111]. The germination energy was calculated at the 5th day. Three independent replicates were performed.

### 4.5. Data Analysis

The one-way analysis of variance (ANOVA) method was employed to statistically analyze experimental data using GraphPad PRISM 8 version 8.0.2 (GraphPad Software Inc., San Diego, CA, USA). In all analyses, *p* < 0.05 was taken to indicate statistical significance and all data are displayed as means ± SD.

## 5. Conclusions

In conclusion, in the present study, we showed that the mutation of *OsPIN1b* substantially impairs rice chilling and drought tolerance. The declined chilling tolerance was evidenced by decreased survival rate, increased cell death cell and increased ion leakage under chilling conditions, which is, at least in part, attributed to the disruption of ROS homeostasis and the suppression of *DREB* genes in *ospin1b* mutants. Additionally, *ospin1b* mutants are more sensitive to drought stress, which is probably caused by the perturbation of ABA homeostasis in *ospin1b* mutants. These results indicate that *OsPIN1b* acts as a pivotal auxin transporter involved in the regulation of chilling and drought adaptation, while the detailed regulatory mechanisms, such as the crosstalk between auxin and ROS, as well as the interaction between auxin and ABA, need further investigation. More experiments, such as an phytohormones assay, and transcriptome and transgenetic analysis, will contribute to investigating the underlying mechanism and will provide new insights for breeding desirable crops with improved abiotic stress tolerance.

## Data Availability

Data are contained within the article and supplementary materials.

## Supplementary Materials

The following supporting information can be downloaded at: https://www.mdpi.com/article/10.3390/plants12234058/s1, Figure S1: The responses of wild-type and *ospin1b* mutants to PEG6000 treatment.; Figure S2: Relative shoot height and relative root length analysis upon PEG6000 treatment. Table S1: Primers used in this study; Table S2: Gene names and ID numbers used for qRT-PCR in this study.
