# Supplementary material for: Deficiency of Auxin Efflux Carrier OsPIN1b Impairs Chilling and Drought Tolerance in Rice
Source: Plants (Basel). 2023 Dec 2;12(23):4058. doi: 10.3390/plants12234058 (PMC10707939; doi:10.3390/plants12234058)

Figure S1. The responses of wild-type and *ospin1b* mutants to PEG6000 treatment. Phenotype, shoot height and root length under normal (A) and drought (B) conditions. Data are means ± SD. Statistically great significant differences ($p < 0.001$) are indicated by three asterisks (***).

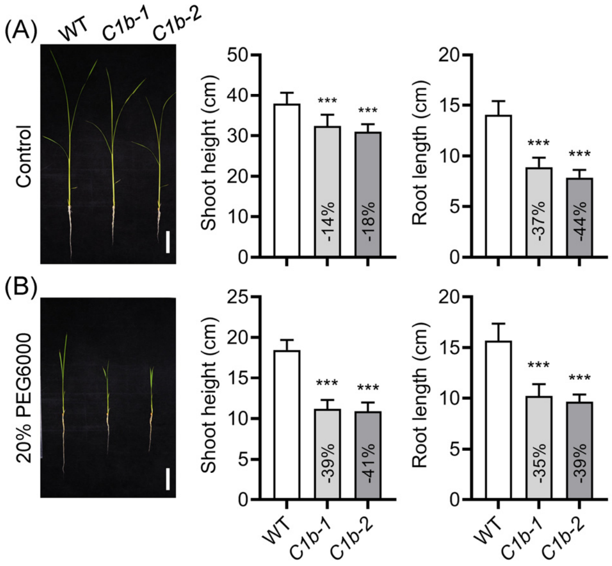

Supplement: Supplementary file 1 [file plants-12-04058-s001.zip › Supplementary files-Figure S1.pdf]
