# Supplementary material for: Deficiency of Auxin Efflux Carrier OsPIN1b Impairs Chilling and Drought Tolerance in Rice
Source: Plants (Basel). 2023 Dec 2;12(23):4058. doi: 10.3390/plants12234058 (PMC10707939; doi:10.3390/plants12234058)

Figure S2. Relative shoot height and relative root length analysis upon PEG6000 treatment. Data were calculated using PEG6000 untreated as a control. Data are means ± SD. Statistically great significant differences ($p < 0.001$) are indicated by three asterisks (***).

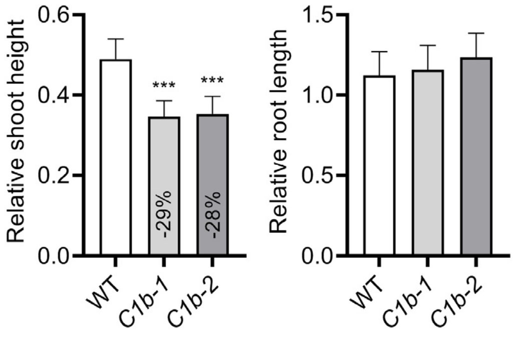

Supplement: Supplementary file 1 [file plants-12-04058-s001.zip › Supplementary files-Figure S2.pdf]
