# Supplementary material for: Deficiency of Auxin Efflux Carrier OsPIN1b Impairs Chilling and Drought Tolerance in Rice
Source: Plants (Basel). 2023 Dec 2;12(23):4058. doi: 10.3390/plants12234058 (PMC10707939; doi:10.3390/plants12234058)
Supplement: Supplementary file 1 [file plants-12-04058-s001.zip › Supplementary files-Table S1.pdf]

Table S1. Primers used in this study.

**Primers for qRT-PCR**	Primer sequences (5′-3′)
OsPIN1a-qF	CCTGAAATCCATCTCCATCCTC
OsPIN1a-qR	AACGTCGCCACCTTGTT
OsPIN1b-qF	GAATCGTGCCCTTTGTGTTTG
OsPIN1b-qR	TGTAGTAGACGAGGGTGATAGG
OsPIN5a-qF	CCCTACCTCAATCCATCACATC
OsPIN5a-qR	GTAGGGAGACAAGCATTCCAA
OsPIN5b-qF	GCAAAGGAGTATGGGCTTCA
OsPIN5b-qR	GCAATCAGAATCGGCAGAGA
OsPIN9-qF	GAGGACTCTCTGTTCACCATTC
OsPIN9-qR	GAGAACGACGCTATCTTGTATCC
OsPIN10a-qF	GTCGAGAAGTCCATCTCCATTC
OsPIN10a-qR	TTGCCACACGCGATGAT
OsYUC1-qF	AGGTGTTGGTCGTGGGATGCG
OsYUC1-qR	GCGATGCCGAACGTGGATAGA
OsYUC3-qF	GGAAGCGTGTTCTCGTTGTTG
OsYUC3-qR	ACATTGACAGCCCAAAGGTGG
OsYUC4-qF	CCTCGACCTCTGCAACCACAATG
OsYUC4-qR	CGACAACAGGAGTACCAGCCAATC
OsYUC5-qF	GTCAGCCTCGACCTCTGCAACA
OsYUC5-qR	TGGGAAACCACTTGAGAAGGAACAC
OsYUC7-qF	ACCGGCTACCGCAGCAATGTG
OsYUC7-qR	CGTACAGCCCCGACTCACCCT
OsYUC8-qF	GAGATGTGCCTGGACCTCTGC
OsYUC8-qR	GTGTCTCCCAGCACCATCCTT
OsDREB1A-qF	AGCGACCTGGCGTTCG
OsDREB1A-qR	TCGCGTAGTACAGGTCCCA
OsDREB1B-qF	GAGACCTTCGCCAACGATG
OsDREB1B-qR	CACCGGCAACACGTCCTT
OsPP2C27-qF	CACTTGGCGGCTATGTT
Os PP2C27-qR	CCTGGCTCACCCACTTC
OsTPP1-qF	TGTCTCCCGTGATGAGAGCTG
OsTPP1-qR	AAACACCTTATTGCGGGACCTT
OsNCED1-qF	CTCACCATGAAGTCCATGAGGCTT
OsNCED1-qR	GTTCTCGTAGTCTTGGTCTTGGCT
OsNCED2-qF	GGTATGGAAACGAGGATAGTGGTT
OsNCED2-qR	TGCTTATTGTTGTGCGAGAAGTTC
OsNCED3-qF	CCCCTCCCAAACCATCCAAACCGA
OsNCED3-qR	TGTGAGCATATCCTGGCGTCGTGA
OsNCED4-qF	TCCATCTCCTTCTCCCTCCTCCCA
OsNCED4-qR	CCTCGCACCCTGCTTGATCTTGCC

OsNCED5-qF	ACATCCGAGCTCCTCGTCGTGAA
OsNCED5-qR	TTGGAAGGTGTTTTGGAATGAACCA
OsPYL1-qF	CAGAGGAAAAAGAAGGCAACGAC
OsPYL1-qR	GCACCACGGTGGAGAAGCA
OsPYL3-qF	AAGGGAACATTGAGATTGGC
OsPYL3-qR	CGGTCAGGATGGAGGAGTAA
OsPYL5-qF	CATCCTCAGCGTCAAGTTCG
OsPYL5-qR	TCACAAGCGTCCCTGGTCT
OsPYL6-qF	ATGATGCCGTACACCGCTCC
OsPYL6-qR	CGCCGCCTTCAACACTCC
OsACTIN1-qF	CTTCATAGGAATGGAAGCTGCG
OsACTIN1-qR	CACCTTGATCTTCATGCTGCTA
