# Supplementary material for: Deficiency of Auxin Efflux Carrier OsPIN1b Impairs Chilling and Drought Tolerance in Rice
Source: Plants (Basel). 2023 Dec 2;12(23):4058. doi: 10.3390/plants12234058 (PMC10707939; doi:10.3390/plants12234058)
Supplement: Supplementary file 1 [file plants-12-04058-s001.zip › Supplementary files-Table S2.pdf]

Table S2. Gene names and ID numbers used for qRT-PCR in this study.

Gene name	Gene ID
OsPIN1a	Os06g0232300
OsPIN1b	Os02g0743400
OsPIN5a	Os01g0919800
OsPIN5b	Os08g0529000
OsPIN9	Os01g0802700
OsPIN10a	Os01g0643300
OsYUC1	Os01g0645400
OsYUC3	Os01g0732700
OsYUC4	Os01g0224700
OsYUC5	Os12g0512000
OsYUC7	Os04g0128900
OsYUC8	Os03g0162000
OsDREB1A	Os09g0522200
OsDREB1B	Os09g0522000
OsPP2C27	Os02g0799000
OsTPP1	Os02g0661100
OsNCED1	Os02g0704000
OsNCED2	Os12g0435200
OsNCED3	Os03g0645900
OsNCED4	Os07g0154100
OsNCED5	Os12g0617400
OsPYL1	Os01g0827800
OsPYL3	Os02g0255500
OsPYL5	Os05g0213500
OsPYL6	Os05g0473000
OsACTIN1	Os03g0718100
